# Context Recognition Algorithms for Energy-Efficient Freezing-of-Gait Detection in Parkinson’s Disease

**DOI:** 10.3390/s23094426

**Published:** 2023-04-30

**Authors:** Luigi Borzì, Luis Sigcha, Gabriella Olmo

**Affiliations:** 1 Data Analytics and Technologies for Health Lab (ANTHEA), Department of Control and Computer Engineering, Politecnico di Torino, 10129 Turin, Italy; gabriella.olmo@polito.it; 2 Data-Driven Computer Engineering (D^2^iCE) Group, Department of Electronic and Computer Engineering, University of Limerick, V94 T9PX Limerick, Ireland; luis.sigcha@ul.ie

**Keywords:** Parkinson’s disease, freezing of gait (FoG), wearable sensors, accelerometer, machine learning, convolutional neural network, random forest, human activity recognition, context awareness

## Abstract

Freezing of gait (FoG) is a disabling clinical phenomenon of Parkinson’s disease (PD) characterized by the inability to move the feet forward despite the intention to walk. It is one of the most troublesome symptoms of PD, leading to an increased risk of falls and reduced quality of life. The combination of wearable inertial sensors and machine learning (ML) algorithms represents a feasible solution to monitor FoG in real-world scenarios. However, traditional FoG detection algorithms process all data indiscriminately without considering the context of the activity during which FoG occurs. This study aimed to develop a lightweight, context-aware algorithm that can activate FoG detection systems only under certain circumstances, thus reducing the computational burden. Several approaches were implemented, including ML and deep learning (DL) gait recognition methods, as well as a single-threshold method based on acceleration magnitude. To train and evaluate the context algorithms, data from a single inertial sensor were extracted using three different datasets encompassing a total of eighty-one PD patients. Sensitivity and specificity for gait recognition ranged from 0.95 to 0.96 and 0.80 to 0.93, respectively, with the one-dimensional convolutional neural network providing the best results. The threshold approach performed better than ML- and DL-based methods when evaluating the effect of context awareness on FoG detection performance. Overall, context algorithms allow for discarding more than 55% of non-FoG data and less than 4% of FoG episodes. The results indicate that a context classifier can reduce the computational burden of FoG detection algorithms without significantly affecting the FoG detection rate. Thus, implementation of context awareness can present an energy-efficient solution for long-term FoG monitoring in ambulatory and free-living settings.

## 1. Introduction

Freezing of gait (FoG) is a disabling motor symptom of Parkinson’s disease (PD) that occurs in more than a half of patients [1]. It consists of a sudden motor block, described by patients as the sensation of having their feet glued to the ground [2]. It is a heterogeneous phenomenon that varies in duration from a few seconds to some minutes, with half of episodes lasting less than 5 s [3]. In addition, it may manifest in different forms, such as shuffling steps, trembling legs, or complete akinesia [4,5,6]. Several circumstances increase the risk of FoG manifestation. For instance, both the number of episodes and their duration increase as the effect of drug therapy decreases [7]. Furthermore, the phenomenon occurs more often during turning, whereas it is less frequent during gait initiation, when approaching the destination, and during straight walking [5]. Certain situations can increase the risk of FoG occurrence, such as stress and both cognitive and motor dual tasks [8,9]. FoG severity increases with disease progression and can lead to falls, injury, loss of independence, and decreased quality of life [10,11]. Hence, continuous monitoring of this phenomenon is crucial to gain information regarding the progress of the disease and the effectiveness of therapy and to estimate the risk of falls [12,13]. At the same time, studying FoG is difficult for several reasons. Its sporadic nature depends on several factors, including pharmacological conditions, attention, triggering situations, and environmental factors. Currently, the clinical assessment of FoG is mainly based on questionnaires administered to the patient and evaluations performed by neurologists in outpatient settings [14]. The former can be subjective and unreliable [15], while the latter consists of a qualitative assessment of the number of episodes, their duration, and the triggering circumstances [16]. However, this assessment during brief and sporadic outpatient visits does not necessarily correspond to a true representation of the phenomenon in daily life.

### 1.1. Technologies for the Automatic Recognition of FoG

Many technological solutions have been proposed to assess FoG continuously and objectively. Optoelectronic systems represent the gold standard for analyzing human movement. These systems require the mounting of many markers on the subject’s body; a set of cameras records the positions of markers in space, thus allowing for an accurate reconstruction of the movement of the entire human body [17]. At the same time, they are expensive and require a long setup time, and the measurements can only be performed in a laboratory. Solutions based on the exclusive use of RGB cameras have been proposed [18,19]. These do not require the mounting of markers. From every single frame, the skeleton is extracted by identifying given pivot points (e.g., the subject’s joints), thus allowing for the evaluation of movements of each limb in real time. However, these solutions require the patient to stay within the range of the camera and are not suitable for continuous unsupervised monitoring.

Finally, motion sensors (e.g., accelerometers and gyroscopes) are small and lightweight and hence can be worn by the patient in daily life. They are inexpensive and allow accurate assessment of human movement in the laboratory, at home, and outside of the house [20]. In recent decades, wearable inertial sensors have been used for a large number of medical applications [21], including FoG monitoring. In this context, many solutions have been proposed based on one or more sensors positioned on different parts of the body to record motion data and automatically detect FoG [22]. However, the accuracy of FoG detection strongly depends on the efficacy of the addressed data processing approaches [23]. For this reason, increasing attention has been devoted over the past decades to the development of effective algorithmic approaches for the automatic identification of FoG [13].

### 1.2. Computer Methods for FoG Detection

The first attempts to recognize FoG using inertial sensor data were based on simple threshold methods. Specifically, an increase in the signal amplitude in the 3–8 Hz *freeze band* was observed during FoG episodes, while the signal during walking was best represented in the 0–3 Hz *locomotor band*. This important information allowed for the computation of the *freeze index*, expressed as the ratio between the freeze and the locomotor band power [24]. A single threshold on such an index provided good results in laboratory settings, with sensitivity and specificity in the ranges of 0.73–88 and 0.82–92, respectively [25].

With the advancements in artificial intelligence techniques, machine learning (ML) algorithms have been employed for FoG detection, such as support vector machine, k-nearest neighbors, and random forest (RF) [26,27,28]. This yielded improved performance in FoG detection, with sensitivity and specificity up to 0.93 and 0.94, respectively [22]. However, the selection of robust and informative features to train ML models strongly affects the final performance and generalization capability [29].

Recently, the advent of deep learning (DL) has provided new opportunities for the automatic processing of raw data without any feature engineering [30]. The approaches based on DL have gained increasing attention, as they allow modelling the data characteristics and motion patterns that best represent FoG and distinguish it from other actions. A wide variety of solutions have been proposed, including convolutional neural networks (CNNs) [31,32], recurrent neural networks [33,34], transformer networks [35], and deep autoencoders [36,37]. A significant improvement in performance has been recorded, with sensitivity and specificity up to 0.92 and 0.98, respectively [38,39].

Most of the proposed solutions were evaluated in a supervised environment, such as the laboratory setting. In this context, subjects were usually asked to perform a scripted set of gait tasks, including different walking paths and turning angles [37,39,40]. However, this does not represent the complete spectrum of activities that patients perform in a home environment, and there is a risk of performance overestimation. For this reason, some research studies included a set of unscripted activities, such as random walking [32] and simulated activities of daily living (ADLs) [41]. The results suggest that FoG detection performance decreases when evaluating classification algorithms on more complex and heterogeneous data. Finally, very few studies collected data in the home environment under unsupervised or semi-supervised conditions [41,42]. The results confirmed the difficulty of accurately detecting FoG in real-world scenarios [42] and triggered the use of complex DL algorithms for data processing [38]. However, in view of continuous and long-term monitoring, a reduced computational burden remains important to improve the autonomy of standalone wearable systems [43].

### 1.3. Context Awareness in FoG Detection

The classical ML processing pipeline encompasses several steps, including filtering, normalization, feature extraction, and, finally, classification. On the other hand, DL models can work directly on raw data, but consist of several processing layers that include thousands of parameters [44]. To reduce the memory usage and computational complexity of FoG detection algorithms, it is possible to use a reduced number of features and lightweight ML models [42,45] or reduce the number of processing layers and parameters in DL algorithms [46]. An alternative approach can exploit the inherent circumstances of FoG manifestation. In fact, FoG mainly occurs during walking and turning. Therefore, activating the FoG detection system only when gait is recognized can help reduce unnecessary computation, thus limiting battery consumption and increasing the energy autonomy of wearable devices. In addition, gait produces inertial signals with a predefined repetitive pattern, which may be easier to recognize than FoG itself. Therefore, simpler methods can likely be used for gait detection, while FoG detection algorithms can be activated only under certain circumstances.

In a previous study [46], we performed a preliminary evaluation of the effect of activity thresholding on FoG detection performance at the window level (i.e., 2 s sliding windows). The proposed method allowed the rejection of up to 40% of windows, slightly improving specificity and significantly reducing sensitivity. However, despite these results, the study was conducted on a single FoG database, and the effects on the number of predicted and detected FoG episodes and computational complexity were not thoroughly evaluated. Furthermore, the proposed approach was not compared with other ML and DL classification models. For these reasons, the present study performs an in-depth evaluation of the effect of context awareness on FoG detection. This was done using different algorithmic approaches and datasets with the aim of reporting new evidence on the effect of context awareness on FoG detection.

### 1.4. Significance of the Study

Although wearable devices can enable long-term monitoring, standard FoG sensing systems and algorithms are designed to analyze entire periods of data and do not consider the context of a subject’s activities. The inclusion of context awareness in prediction pipelines can help alleviate the computational load produced by accurate (and generally complex) algorithms. Furthermore, context information can be used to prevent the generation of false positives in FoG detection produced by the execution of walking-like activities. For this reason, the present study aims to develop an efficient context algorithm to reduce the computational burden of FoG detection algorithms and increase the autonomy of monitoring systems based on wearable devices. The main contributions of this work can be summarized as follows.

- Different algorithmic approaches with different complexity levels are compared. A simple threshold method based on signal magnitude is used to distinguish activity from inactivity periods. Classic ML approaches are implemented, using temporal and spectral features to feed two ML classifiers. Finally, a DL model is implemented and evaluated using raw acceleration data.
- The performance of various gait detection algorithms is evaluated and compared on a dataset that includes gait and different ADLs.
- The effect of context algorithms on FoG detection is evaluated using two datasets including FoG, different walking tasks, and ADLs.
- The computational complexity and testing time are evaluated and compared between the approaches and with related studies.

The remainder of this paper is organized as follows. Section 2 describes the data and processing methods to develop context algorithms for FoG detection, as well as performance evaluation procedures. Results are reported in Section 3 and discussed in Section 4. Finally, conclusions are drawn in Section 5, along with a discussion of future work.

## 2. Materials and Methods

This section describes the materials and methods used in this study. More specifically, Section 2.1 provides an overview of the data used in this study, the implemented algorithms, and the outcomes. Section 2.2 describes the three databases employed to test the algorithms. Data preprocessing procedures are reported in Section 2.3. Section 2.4 describes the ML and DL methods implemented for gait detection, along with the simple threshold approach. Performance evaluation procedures and performance metrics are discussed in Section 2.5, while Section 2.6 reports the methods for assessing the effect of context information on FoG detection performance.

### 2.1. Proposed Framework

**Figure 1:**
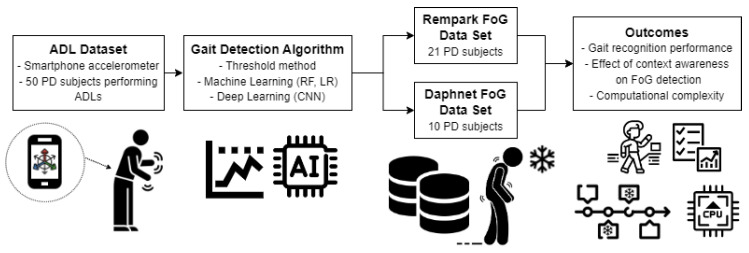
Proposed framework that uses context recognition algorithms for FoG detection. RF: random forest; LR: logistic regression; CNN: convolutional neural network; PD: Parkinson’s disease; FoG: freezing of gait.

In the proposed framework, the effect of gait recognition algorithms applied to FoG detection is evaluated. Three datasets were used for this analysis. Among them, the first dataset (ADL) was used to implement and evaluate the performance of different algorithmic approaches for gait detection. Subsequently, gait detection was used to provide context awareness before the implementation of FoG detection algorithms. Finally, the other two datasets (Rempark and Daphnet) were used as benchmarks to evaluate the effects of using a gait detection algorithm prior to FoG detection. The latter evaluation includes analysis of the percentage of predicted and detected episodes. Furthermore, computational complexity was evaluated and compared between the approaches. A schematic representation of the proposed framework is provided in Figure 1.

### 2.2. Data

Three databases were used in this study, including a different number of patients with PD (PwPD) performing different sets of activities. Data from a single inertial sensor placed on the lower back were extracted and employed for the subsequent analysis. Specifically, the ADL dataset was used to train the gait detection algorithms and evaluate their performance. On the other hand, the Rempark and Daphnet datasets were used to evaluate the effect of context algorithms on both FoG detection and computational complexity reduction. More detailed information about the datasets is provided in the following.

**ADL dataset**. The dataset [47] utilized in this study comprises data from fifty-nine PwPD. Inclusion criteria required a clinical diagnosis of PD with motor symptoms, with or without a medical history of FoG events, and no significant comorbidities or impairments in vision/cognition that would hinder task performance. Participants who required gait assistance aids such as walking sticks or crutches were included. Data collection took place during pre-scheduled outpatient visits, with all participants in a daily ON state, having taken their usual medication dose with variable amounts time elapsed since then. Nine PwPD were excluded from subsequent analysis due to minimal or no gait activity, resulting in a total of 50 PwPD included in this study. The sample consisted of 32 males and 18 females, with an average age of 70.9 ± 9.8 years, disease duration of 7.2 ± 5.4 years, Hoehn and Yahr (H&Y) score of 2.3 ± 0.8, and a total Unified Parkinson’s Disease Rating Scale (UPDRS) part-III score of 30.7 ± 11.2. Data from a three-axis accelerometer and a three-axis gyroscope were recorded using a smartphone attached to the lower back using an elastic band. The accelerometer and gyroscope were set to a range of ±2 g and ±2000 dps, respectively, with a sampling rate of 200 Hz. Inertial data were stored locally on the smartphone. Data collection was conducted during outpatient visits, and participants were instructed by clinicians to perform various activities, including free walking, standing up, sitting down, sitting and standing for several seconds, turning with different angular amplitudes, and other tasks assessed during the UPDRS evaluation. These tasks were intended to represent the activities typically performed in a domestic environment. In total, 7.4 h of inertial data were recorded, including 28.3 min of gait, 27.5 min of stance (i.e., sitting and standing), 13.4 min of postural transitions (i.e., sitting down and standing up), and 18.6 min of UPDRS-related activities (e.g., toe-tapping, leg agility, pull test, and finger to nose), while the remaining activities included other scripted tasks (e.g., taking a book from the library, putting it on a desk, and returning it to the library) and unlabeled activities.
**Rempark dataset**. The dataset [41] comprises data from twenty-one individuals who were clinically diagnosed with PD and had motor symptoms. To be included in the dataset, participants had to have an H&Y stage greater than 2 in the OFF state of therapy, a FoG questionnaire (FoG-Q) score greater than 6, and no vision impairments or dementia that would impede their ability to complete the required tasks. Participants who required assistance while walking were still included in the study. The experiments were conducted in the participants’ homes, and data were collected both while the participants were ON and OFF dopaminergic therapy. The sample consisted of three women and eighteen men, with an average age of 69.3 ± 9.7. The participants had a disease duration of 9 ± 4.8 years, an H&Y score of 3.1 ± 0.4, a FoG-Q score of 15.8 ± 4.1, a mini-mental state examination score of 27.8 ± 1.9, and a total UPDRS part-III score of 16.2 ± 9.7 ON and 36.3 ± 14.4 OFF therapy. The tasks performed included gait tasks such as walking outdoors, the stand-up-and-go test, and showing the participant’s home. Additionally, false positive analysis tasks such as cleaning windows, brushing teeth, and painting/drawing/erasing on a sheet of paper were considered for the study. For data collection, an inertial measurement unit (IMU) was attached to the left side of the waist using an elastic band to record three-axis acceleration data, which were stored on the device memory. The accelerometer range was set to ±6 g, and data were sampled at a rate of 200 Hz, which was later down-sampled to 40 Hz. During the experiments, a total of 9.1 h of inertial data were recorded, including 93 min of FoG.
**Daphnet dataset**. The dataset [25] comprises data from ten PwPD. In order to be included, participants had to have a clinical diagnosis of PD and a history of FoG, be able to walk unassisted in the OFF therapy state, and have no severe vision or hearing loss, dementia, or other neurological/orthopedic diseases. Experiments took place in the morning during the OFF stage of the medication cycle, which was more than 12 h after their last drug intake. Two participants who reported frequent FoG episodes during the ON state were not asked to avoid taking medication. Participants were asked to complete three walking tasks that aimed to represent different aspects of daily walking. These tasks included walking forth and back in a straight line along the lab hallway and random walking in a reception hall space with initiated stops and 360-degree turns. In addition, walking while simulating ADLs was considered in the protocol, including entry and exit of rooms and walking to the lab kitchen, getting a drink, and returning to the starting room with a cup of water. The sample consisted of seven males and three females, with an average age of 66.4 ± 4.8 years, a disease duration of 13.7 ± 9.7 years, and an H&Y score of 2.6 ± 0.65 in ON conditions. During the experiments, data from three accelerometers placed on the shank, thigh, and lower back were recorded at a sampling rate of 64 Hz. A total of 4.9 h of inertial data was recorded, including 28.9 min of FoG.

A summary of the databases used in this study is reported in Table 1. For each dataset, information is provided on the sample, wearable device and embedded sensors, and the position of sensors on the body.

### 2.3. Pre-Processing

As reported in Table 2, data from the different datasets were recorded using different sensor settings. In particular, different sensor orientations, measurement units, and sampling rates were employed. To standardize the data, the acceleration recordings were resampled at 40 Hz and converted to g-unit. In addition, the readings were arranged so that the *x*-, *y*- and *z*-axes represented the anterior, vertical (downward), and lateral (rightward) directions, respectively.

**Figure 2:**
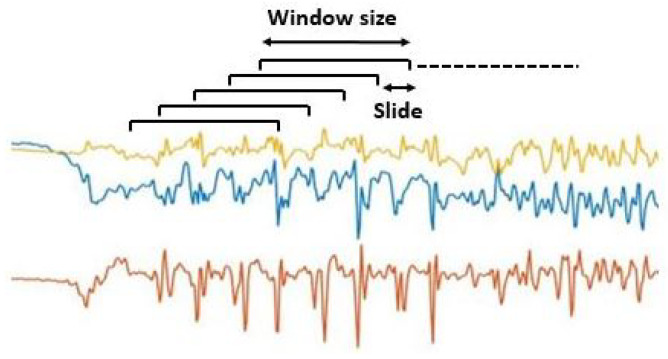
Segmentation process performed on raw acceleration data using windows of 2 s sliding with a step of 0.5 s.

No further filtering or normalization procedures were performed on raw data. Instead, the acceleration signals were segmented using fixed-length windows of 2 s (i.e., 80 samples) sliding with a step of 0.5 s (i.e., 75% overlap) according to Figure 2. Additionally, the average value was removed from every single window separately, as was also done in [46]. This strategy allows one to work independently on each window, thus making the algorithm suitable for real-time implementation. In order to train the gait recognition algorithms described in the following sections, the ADL dataset labels were organized into two classes (i.e., gait or non-gait) to set up a binary classification problem. Specifically, the walking and turning labels were grouped to form the gait class. All other activities, including static postures (i.e., standing and sitting), postural transitions (i.e., standing up and sitting down), and activities related to clinical assessment (e.g., pull tests, leg agility, toe-tapping, and upper limb movements) were grouped together to form the non-gait class.

### 2.4. Gait Recognition for Context Awareness

Different approaches were implemented for context recognition. A classic ML processing pipeline is described in Section 2.4.1, while the implementation of a more complex DL classification algorithm is reported in Section 2.4.2. Finally, a simple threshold method based on activity intensity is described in Section 2.4.3.

#### 2.4.1. Machine Learning Algorithms

Classic ML pipelines require the extraction of discriminant features from raw signals. In this study, a set of features extracted from both the time and the frequency domains was used. The features from the time domain yield a high discriminative capability without involving a significant increase in computational complexity [27]. On the other hand, the features from the frequency domain are useful to describe repetitive motion patterns [48]. A total of 42 temporal features (14 per channel) and 33 spectral features (11 per channel) were extracted, leading to a total number of 75 features. A summary of the feature set used in this study is shown in Table 3. Some of these features represent basic characteristics describing the amplitude of the signal (e.g., median, range, minimum, and maximum values). Other more complex features were previously used for walking (e.g., maximum spectral peak height, frequency of the dominant harmonic, and width of the dominant harmonic) [49] and turning (e.g., jerk, spectral entropy, and power density in the postural band) [47] analysis, while others were used for FoG detection (e.g., increments, principal components, kurtosis, and skewness) [41,50]. This set of features was used to feed two ML classification algorithms, including a logistic regression (LR) model and an RF classifier [51] with 100 estimators. Additional model parameters include a minimum sample split equal to 2, a minimum sample leaf equal to 1, a maximum depth of the tree equal to 3, and the split criterion set to Gini impurity [52]. Most of the hyperparameters (e.g., number of estimators, minimum sample split, minimum sample leaf, maximum depth of the tree) were selected using a grid-search tuning process; however, computational complexity was taken into account, with the aim of ensuring a low computational burden without significantly impairing the performance.

Furthermore, the synthetic minority oversampling technique (SMOTE) [53] was used to balance input data, with a number of nearest neighbors equal to 5. This technique has been used in FoG detection tasks, demonstrating increased detection performance compared to an unbalanced dataset and providing better results than undersampling and oversampling methods [54]. Specifically, the class with a minority number of feature vectors (i.e., gait: 3490 feature vectors (8%)) was resampled to provide the same number of feature vectors as the majority class (i.e., non-gait: 46,558 feature vectors (92%)). The data augmentation technique described was applied exclusively to the training subset, with no modifications made to the testing subset.

#### 2.4.2. Deep Learning Model

In this work, a DL classification algorithm was implemented using one-dimensional (1D) CNN layers. The CNN architecture encompasses two convolutional layers with pooling operations that are connected to a single fully connected output layer. The implemented architecture represents a modified version of that proposed by Bikas et al. [32]. However, to reduce the complexity of the model, separable 1D convolution layers [55] were used instead of standard convolutional layers. Specifically, separable convolutions implement a depthwise spatial convolution, which acts separately on each input channel, followed by a point-wise convolution that mixes the resulting output channels. Raw acceleration recordings were input to the 1D CNN, exploiting the capability of the convolutional layers to automatically extract a set of discriminant features [56].

**Figure 3:**
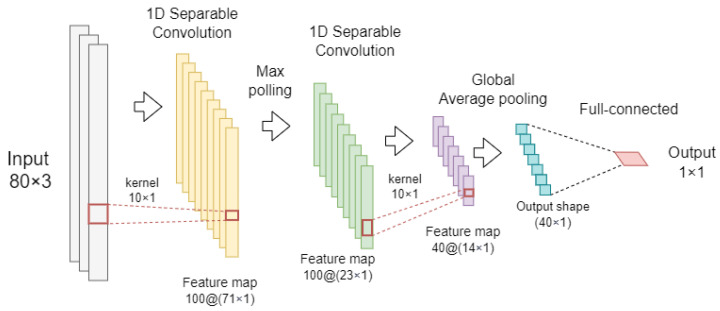
Proposed architecture of the 1D CNN with separable convolutions.

The proposed CNN architecture with separable 1D convolution layers (1D SepConv CNN) is schematically reported in Figure 3. Specifically, the CNN consists of an input layer with a dimension of 80 samples and 3 channels (e.g., acceleration signals along the x, y, and z directions). The input layer is connected to two 1D separable convolutions layers, both with the ReLU activation function. The first convolutional layer has 100 filters with a kernel size of 10 and is connected to a max pooling layer with a pool size equal to 3. The second convolutional layer has 40 filters with a kernel size of 10 and is connected to a global average pooling layer (GAP) that outputs a one-dimensional feature map. For the classification task, a fully connected layer was used to generate the prediction output. This output layer uses a single neuron with a sigmoid activation function. Additionally, a dropout of 0.5 was applied before the fully connected layer to prevent overfitting. Table 4 reports a summary of the parameters used in the CNN network with 1D separable convolution.

Hyperparameter optimization of the CNN was performed using the hyperband method [57]. In this process, the learning rate, weight decay, and batch size were optimized. The model was trained using an adaptive moment estimation optimizer with decoupled weight decay (AdamW) [58], learning rate $1\cdot 10^{-3}$, and weight decay $3\cdot 10^{-5}$. Furthermore, the binary cross-entropy loss function, a batch size of 512, and a maximum number of iterations of 300 were used for training the CNN. Additionally, an early stop condition was implemented to avoid overfitting and unnecessary calculations during training. This strategy stops training when the validation loss does not decrease across 10 continuous epochs.

#### 2.4.3. Threshold Approach

**Figure 4:**
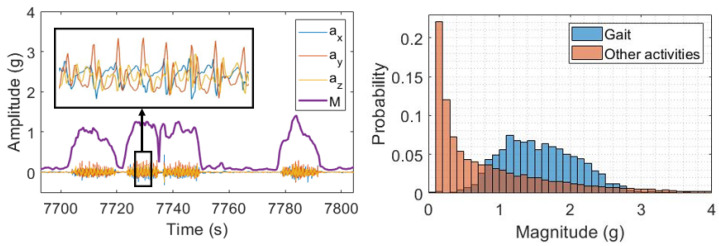
(**Left**) The 3D acceleration signals along with the resulting magnitude vector (violet). A zoomed image of gait signals is also reported. (**Right**) Histograms reporting the distribution of the magnitude vector values for gait and other activities. Data represents the ADL dataset.

The threshold approach implemented in this work aims to distinguish between periods of activity and inactivity using a simple thresholding operation. To this end, the magnitude $M_{j}$ of the 3D acceleration signal for each window *j* was calculated according to Equation (1), where $\alpha_{x}$, $\alpha_{y}$, and $\alpha_{z}$ represent the acceleration signals along each axis, respectively, and the sum is performed for each sample *i* of each window of length *w*. Figure 4 (left) shows a segment of acceleration recordings from the ADL dataset, along with the resulting magnitude vector. A zoomed segment of acceleration signals during gait is also reported.

(1) $$M_{j}=\sqrt{\sum_{i=1}^{w}\left({\alpha_{x_{i}}}^{2}+\alpha_{y}{{}_{i}}^{2}+\alpha_{z}{{}_{i}}^{2}\right)}$$

The magnitude vector was computed for all datasets used in this study. In order to identify the threshold that allows the identification of most gait windows while discarding other activities, the following procedure was performed. The ADL dataset was used to fine-tune the threshold. Specifically, the distribution of magnitude values was compared between gait and other activities, as reported in the histogram in Figure 4 (right). As can be observed, the magnitude values partially overlap between gait and other activities. Thus, the threshold selection represents a trade-off between the number of gait windows detected and the number of non-gait windows discarded. A fine-tuning procedure was set, varying the threshold in the 0–1 g range and computing the F-score (see Section 2.5) for gait detection. The value corresponding to the maximum F-score was selected as the final threshold.

### 2.5. Evaluation Methodology and Performance Evaluation

In order to provide a comprehensive performance evaluation and properly assess the generalization capability of the developed algorithms, the following procedures were performed. The ADL dataset was divided into training, validation, and test subsets with proportions of 50% (25 PwPD), 25% (12 PwPD), and 25% (13 PwPD), respectively. On the other hand, data from the Rempark and Daphnet datasets were not further processed, as they were used as independent test sets.

The gait recognition algorithms were then trained on the training subset, optimized using the validation subset, and tested on the test subset. Data from each subject entered a single subset exclusively, thus ensuring subject independence. This data-splitting strategy avoids overfitting and promotes the generalization capability of the resulting classification model. Finally, the following metrics were calculated to evaluate the algorithm performance in recognizing gait. Sensitivity (Equation (2)) represents the algorithm’s capability of detecting true positive (TP) samples while discarding false negatives (FP). Specificity (Equation (2)) represents the capability of recognizing true negative samples (TN) while discarding false positives (FP).

(2) $$Sensitivity=\frac{TP}{TP+FN}\;\;\;\;\;\;\;\;\;\;\;\;\;\;\;\;\;\;\;\;Specificity=\frac{TN}{TN+FP}$$

F-score (Equation (3)) is the harmonic mean of sensitivity and precision (Equation (4)). In the case of unbalanced datasets, F-score is preferred to accuracy as the global classification metric.

(3) $$F-score=\frac{2\cdot Sensitivity\cdot Precision}{Sensitivity+Precision}$$

(4) $$Precision=\frac{TP}{TP+FP}$$

To provide further comparison, both the area under the receiver operating characteristic (AUROC) and the equal error rate (EER) were computed. AUROC evaluates a classifier’s capability to differentiate between classes and serves as a summary of the ROC curve, while EER represents the error rate observed at the point on the ROC curve where sensitivity equals specificity.

### 2.6. Effect of Context Awareness on FoG Detection

**Figure 5:**
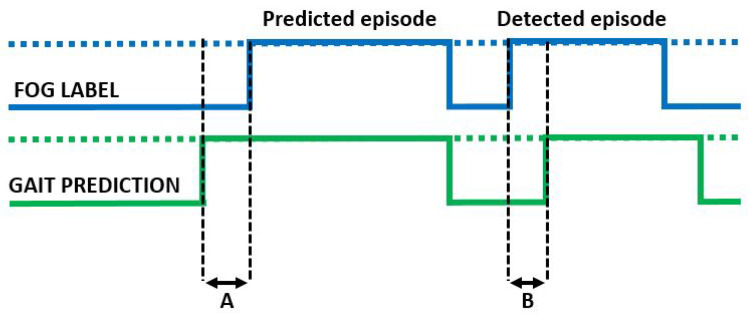
Schematic of the measures computed for evaluating the performance of the gait recognition approaches for context-aware FoG detection. A: activation horizon; B: activation delay.

To evaluate the effect of the context algorithms on FoG detection, the gait detection approaches described in Section 2.4 were tested on the Rempark and Daphnet datasets separately. For each model, the gait/activity predictions were compared to the FoG label available from the FoG datasets. Figure 5 schematically reports the performance metrics used in this study to evaluate the effect of context algorithms on FoG detection. On the one hand, when gait/activity was recognized before FoG, the episode was considered predicted. In this case, the activation horizon was computed as the difference between the first gait-detected window and the actual FoG onset (Figure 5A). On the other hand, when gait was identified after the actual FoG onset, the episode was considered detected with a certain delay (Figure 5B). The percentage of predicted and detected episodes was computed, and both the activation horizon and the activation delay were expressed in seconds. When the activation delay was larger than 3 s from FoG onset, the episode was considered not detected. Finally, the time active measure was calculated as the percentage of time in which gait/activity was identified.

Finally, to compare the complexity of the designed context algorithms to that of similar studies, the following measures were calculated. The total number of parameters, the number of floating point operations (FLOPs) to perform the prediction on a single sliding window, and the prediction time were either extracted from the related studies (when reported) or calculated by reproducing the reported models.

The analyses were performed on a computer with a 2.3 GHz processor, 8 GB RAM, and 4 GB GPU. MATLAB (version R2022a) was used for pre-processing and post-processing, whereas Python (version 3.6) was employed for training, optimization, and testing of classification models. In addition, scikit-learn (version 1.2.2), TensorFlow (version 2.3), Keras (version 2.4), and Keras-FLOPs (version 0.1.2) libraries were used to perform the experiments.

## 3. Results

This section reports the results for the different gait recognition algorithms (Section 3.1) and threshold-based context detection (Section 3.2). Moreover, the effect of the context algorithms on FoG detection is reported in Section 3.3.

### 3.1. Gait Recognition Performance

The performance of gait detection algorithms based on the ML and DL approaches is reported in Table 5. To assess the presence of overfitting, performance metrics are reported separately for the training, validation, and test sets. As can be observed, performance slightly decreases from the training to the test set, and the effect is more evident in the F-score and EER metrics. The DL approach shows more consistent performance across sets, as evident from all metrics except the F-score. Overall, the DL-based gait detection performed better than the ML methods (i.e., LR and RF). Specifically, the results on the test set showed a clear increase in specificity (+3.3%) and F-score (+9.3%) and a decrease in EER (−4%), while sensitivity and AUROC were comparable.

### 3.2. Threshold-Based Approach

**Figure 6:**
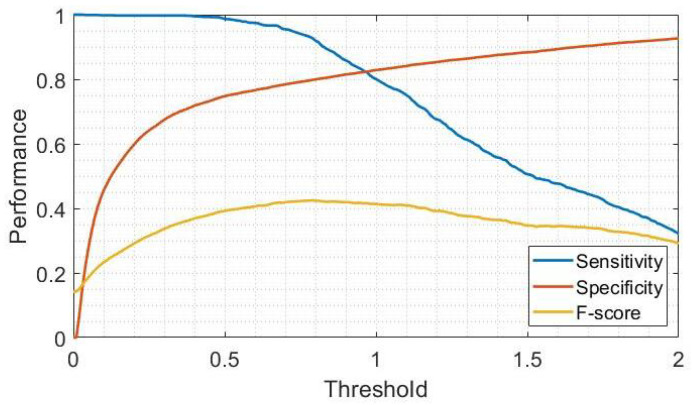
Performance in gait detection as the threshold on the magnitude vector varies.

Figure 6 shows the performance in gait detection as the threshold set on the magnitude vector increases. As can be seen, the decrease in sensitivity is minor up to 0.5, while it becomes evident for higher threshold values. On the other hand, specificity exhibits an exponential increase between 0 and 0.5, while the trend is linear for higher values. Sensitivity and specificity curves cross when the threshold is 0.97. Setting the threshold at this value results in a sensitivity and specificity of 0.822. As for the F-score, it shows a bell-shaped pattern, with the maximum value corresponding to a threshold of 0.8. Using this value as a threshold, the sensitivity and specificity turn out to be 0.926 and 0.801, respectively. From these results, it is evident that the use of the latter threshold allows a marked increase in sensitivity (+10.4%) at the expense of a small reduction in specificity (−2.1%). Therefore, the final threshold was set to 0.8 and was used to test the approach on the two FoG datasets.

### 3.3. Effect of Context Awareness on FoG Detection

Table 6 reports the effect of the four context detection approaches on the Rempark dataset. Results are expressed in terms of the percentage of predicted and detected episodes, activation horizon, and activation delay. In addition, the percentage of time when gait/activity is recognized is provided by the time active metric. As is evident, the ML approaches (i.e., LR and RF) show lower performance than the DL and threshold approaches, as evidenced by the lower percentage of predicted FoG episodes. Performance is similar between the DL and the threshold approach, with 96% and 95% of FoG predicted with an advance of more than 8.2 s and 4% and 5% of FoG detected in less than 0.8 s. However, the latter shows a reduced activation time (−4%), which allows for discarding a larger number of windows.

Table 7 shows the effect of the four context detection approaches on the Daphnet dataset. Again, the DL and threshold methods performed better than the ML models, as shown by the higher percentage of predicted episodes. However, the activation time slightly increases by 3.1–4.4%. The DL and threshold approaches produce similar activation times; however, the latter method provides the best performance, with 94% of FoG episodes predicted. It is worth noting that, in this case, the activation horizon provided by the threshold approach is larger than that obtained by the DL and ML models.

When comparing the effect of the context algorithms on the two different datasets, the following considerations can be made. All approaches show a smaller number of predicted episodes, while longer prediction horizons and total activation times are observed. Specifically, the percentage of predicted episodes decreases by 1–9%, while the increases in the activation horizon and total activation time range from 3.3 s to 12.4 s and from 1.9% to 5.7%, respectively. The threshold approach provided the most consistent results across datasets, with a similar percentage of FoG episodes predicted (difference < 1%) in the two databases. The increase in the total activation time can be partially explained by the fact that there is more activation during the gait preceding the FoG, as evidenced by the increase in the average activation horizon. Finally, while in the Rempark dataset all FoG episodes are predicted or detected within 3 s from their occurrence, 2%, 3%, and 4% of FoG was not detected in a timely manner in the Daphnet dataset using the ML, DL, and threshold approaches, respectively.

### 3.4. Computational Complexity

In the related literature, several DL classification models have been proposed for FoG detection with promising performance. However, their computational burden should be considered. Table 8 describes the computational complexity of some FoG detection algorithms based on DL in terms of number of parameters, number of FLOPs, and prediction time. All reported algorithms were developed for the analysis of inertial data from a single wearable inertial sensor positioned on the lower back. As can be observed, the multi-head CNN model proposed in [46] presents around 10 thousand parameters and requires less than half a million FLOPs to perform a prediction on a single (2 s long) window. This result is comparable to the evaluation of related DL methods such as those proposed in [38] and is significantly lower than those presented in [23,32,35]. The prediction time is similar for all algorithms, ranging from 38 to 45 ms, with the longest time being for the CNN-Transformer. The 1D SepConv CNN model has a significantly lower number of parameters (5.5 K). However, a total of 0.41 M FLOPs are required to make a prediction, which is in line with light DL approaches for detecting FoG [38,46]. Thus, the use of such a model for context awareness can bring significant advantages only when combined with complex DL algorithms, such as those proposed in [23,32,35], while it does not provide any advantage over the DL models proposed in [38,46].

On the other hand, the threshold approach consists of calculation of the root sum of squared acceleration values. Considering a window of data of length *w*, vector products and sums require 2·w and *w* FLOPs, respectively, while root and scalar multiplication require a single FLOP. Thus, only 9·w+2 FLOPs are required in total, with w=2·Fs corresponding to the window size and Fs=40 Hz being the sampling frequency. This leads to a single parameter, i.e., the scalar vector multiplying the magnitude value, and 722 FLOPs. Compared to light DL models (e.g., [38,46]), the energy consumption of the threshold approach provides an energy saving of 98.8% in the data processing stage.

## 4. Discussion

Different gait recognition algorithms were implemented based on classical ML models and a more complex DL method. The latter provided better results in all performance metrics, with a sensitivity of 0.96 and a specificity of 0.93. Moreover, it demonstrated robustness across sets with the absence of overfitting. It is worth considering that the validation and test sets comprised more than twelve patients each, and subject independence was guaranteed. This provides significance to the obtained results and proves the good generalization capability. The threshold approach provided a sensitivity of 0.93 and a specificity of 0.80 in gait detection despite the simple processing method and the fact that this approach was not specifically designed for gait detection.

Testing the context algorithms on two different FoG datasets allowed for the prediction of most FoG episodes while reducing the number of windows to be analyzed by FoG detection systems. In the case of DL and threshold approaches, more than 87% of FoG was predicted before its actual manifestation with a temporal horizon of more than 8 s. However, the threshold approach provided more consistent performance across datasets, with more than 94% FoG predicted both in the Rempark and Daphnet datasets. Moreover, this method represents the ideal solution, given the very reduced computation burden compared to the DL method. It is worth noting that the DL model architecture was very light, with only two convolutional layers and one fully connected layer, along with the use of separable convolutions. Further complexity reduction (e.g., reducing the number of filters) is likely to yield impaired performance. On the other hand, increasing the model complexity (e.g., by adding additional convolutional and/or fully connected layers) can provide better results; however, this would not have the advantages of a lightweight computational method.

The fact that a simple threshold on the acceleration magnitude provided better results than ML and DL approaches can be explained by the degraded gait pattern manifesting just before FoG occurrence. In a previous work on FoG detection [46], more than 50% of FoG episodes were predicted on average 3.1 s before their actual occurrence. This testifies that the gait pattern preceding FoG can be confused with FoG. As a further confirmation, in [35], we found that around 35% of false detections (i.e., activities other than FoG identified as FoG) were less than 5 s distant from real FoG episodes. From these considerations, it becomes clear that gait can be severely impaired as the FoG episode approaches, and gait recognition models may fail to identify such atypical walking patterns.

The implemented context algorithms can activate FoG detection systems in specific situations. On the one hand, the results show that there are very few missed FoG episodes. On the other hand, this allows most of the data to be processed using a very simple method with a low computational load. In fact, complex and computationally expensive FoG detection methods are activated less than 50% of the time. The latter represents a conservative result. In fact, in both the Rempark and Daphnet datasets, data were collected while patients performed a variety of walking tasks in addition to common ADLs. This was done to increase the probability of FoG occurrence. However, in real-world scenarios, the percentage of time patients walk is much lower than that reported in the two FoG datasets. This means that FoG detection systems can be activated much less frequently than observed in the present study. This allows for a significant reduction in data processing and a consequent increase in the battery life of wearable systems.

The developed context algorithm for FoG detection can work nearly in real-time. In fact, the input is a single 2 s long window that is analyzed every 0.5 s. This represents a suitable delay to activate FoG detection systems. As for the computational complexity, only mean removal and magnitude computations are performed on the input window, providing a very efficient and fast signal processing method. The proposed approach can be implemented in real-time applications, enabling the implementation of gait assistance systems. These can reduce the duration and frequency of FoG episodes by providing auditory, visual, or somatosensory feedback cues that help PwPD to maintain the speed and amplitude of their movements [59,60,61,62].

Previous studies have explored the contextualization of activities to perform an automatic assessment of cardinal motor symptoms such as bradykinesia and tremor using both hierarchical [63] and multi-task [64] approaches, and also in FoG detection using fuzzy logic [45]. However, in this study, we propose the evaluation of several algorithmic approaches for context awareness in FoG detection with the aim of identifying a suitable approach for use in long-term monitoring. Based on the results, the use of an activity detection algorithm based on thresholds seems to be an effective solution to implement a context awareness strategy for FoG detection.

In this study, data from a single accelerometer were used for analysis. This limits the generalizability of the results obtained to other body positions. On the one hand, sensors placed on the patients’ lower extremities (e.g., feet, ankles, or shins) provide a better representation of the gait pattern and thus ease the analysis. On the other hand, data recorded by sensors placed on the upper extremities (e.g., wrists) may involve different or more demanding processing. However, it is worth noting that, according to [65], the lower back was considered a comfortable and acceptable position by more than a hundred PwPD who wore a wearable device at home for one week.

This study has some limitations. First, context algorithms may fail to activate FoG detection algorithms in advance of FoG episodes when no gait or activity is detected prior to FoG occurrence. In particular, start hesitation [4] occurs when patients begin to walk and may pose a challenge when preceded by a static position (e.g., stance). Furthermore, akinetic FoG does not produce any observable leg movements, and this may pose a further challenge for the timely activation of FoG detection algorithms. However, start hesitation is less frequent than FoG during turning or walking. Furthermore, FoG during turning might be of greater clinical importance because patients are less stable and the risk of falling might be higher while turning [5]. Finally, PwPD with pure akinetic FoG are rare [6], and akinetic FoG is generally uncommon in PwPD, occurring in about 10% of all episodes [5].

It could be very useful to evaluate the gait recognition performance on the Rempark and Daphnet datasets. This would allow for assessing the real generalization capability of the algorithms when performing cross-dataset testing procedures. However, such datasets provide only a binary label, i.e., FoG or non-FoG, without a specific indication of walking bouts. Finally, as discussed earlier, data from real-life scenarios are necessary to obtain a realistic evaluation of battery savings. The Rempark database comprises data related to activities similar to those of daily living and recorded in the home environment. However, patients were asked to perform several gait and FoG-provoking tasks to increase the probability of FoG occurrence. This increases the proportion of gait data that may not represent normal daily life.

In this study, data analysis was limited to inertial sensor data. However, it would be useful to obtain contextual information from different sources, such as physiological (e.g., heart rate or respiration) and environmental sensors. This could help identify which types of data are most informative for FoG prediction/detection and which can be safely ignored to reduce the computational load. In order to not affect patient comfort, it would be important to use non-invasive technology to record physiological data (e.g., a smartwatch).

Finally, the segmentation process consisted of 2 s fixed-length windows sliding with a step of 0.5 s. This is in line with the real-time FoG detection algorithm proposed in [46], which can be combined with the contextual method developed in this work. However, the use of a larger window and step can increase performance and reduce the computational load [66]. On the other hand, this may negatively affect the temporal resolution in gait and FoG recognition. Future studies could evaluate the effect of varying window size and overlap on performance and battery consumption.

## 5. Conclusions

This study evaluates the effects of contextual algorithms applied to FoG detection. Specifically, four algorithmic approaches with different levels of complexity were designed for the detection of gait and activity. Performance was evaluated on a dataset comprising fifty PwPD performing gait and ADLs. The impact of context awareness on FoG detection performance was assessed in two different datasets comprising thirty-one PwPD performing a large number of walking tasks and ADLs and including more than 1200 FoG episodes. In this work, data recorded by a single accelerometer placed on the lower back were analyzed. This represents a simple and unobtrusive sensor configuration for passive long-term monitoring of PD. The results indicate that the use of a single inertial sensor and the implementation of context-aware approaches appear to be a viable option for implementing ecological and energy-efficient solutions for long-term FoG monitoring in ambulatory and free-living settings.

As FoG episodes can occur several times a day, the development of accurate and optimized algorithms for long-term FoG monitoring remains crucial. Although wearable sensors and smart devices (e.g., smartphones) can enable long-term monitoring during daily life, the implementation of complex (but accurate) algorithms poses a problem for device autonomy. In this study, the implementation of context-awareness in FoG recognition indicates that the use of a threshold (lightweight) approach can help to reduce the computational load produced by the implementation of complex pipelines for FoG detection. With this strategy, FoG recognition algorithms can be activated only during periods when gait is detected in order to reduce battery consumption, thereby increasing the energy autonomy of the monitoring devices. Furthermore, the performance was consistent across the datasets, allowing the majority of non-FoG data to be discarded and less than 4% of FoG episodes to be missed. These results promote the development of generalized solutions that do not depend on the use of a particular device, as demonstrated by the generalization capability of the proposed algorithm to external datasets.

Future work will go in the direction of implementing a context-aware FoG detection algorithm on a standalone wearable system. This can be used for passive collection of activity, gait, and FoG information and to provide real-time feedback to reduce the severity of FoG. Furthermore, context awareness can be exploited in the evaluation of other motor manifestations and can also consider the use of different data modalities (e.g., biosignal, video, multimodal, etc.), assessing the impact of contextualizing specific activities using sensors placed on a different part of the body.

## Figures and Tables

**Table 1:** Description of the databases used in this study. PwPD: patients with Parkinson’s disease; ADLs: activities of daily living; FoG: freezing of gait; IMU: inertial measurement unit.

Database	Description	# Subjects (with FoG)	Device	Sensor Type (# of Sensors)	Sensor Location
ADL [47]	Data collected from PwPD when performing scripted ADLs and UPDRS-related activities. No FoG episodes were recorded.	50 PwPD (0)	Smartphone	Triaxial accelerometer (1), Triaxial gyroscope (1)	Lower back
Rempark [41]	Data collected in the home environment when PwPD performed a set of scripted ADLs. The dataset includes 1058 FoG episodes.	21 PwPD (21)	Prototype IMU	Triaxial accelerometer (1), Triaxial gyroscope (1)	Waist
Daphnet [25]	Gait and FoG measurements collected in the laboratory when PwPD performed walking tasks and ADLs. The dataset includes 237 FoG episodes.	10 PwPD (8)	Wearable sensors	Triaxial accelerometers (3)	Lower back, upper-leg, lower-leg

**Table 2:** Data recording settings in the different datasets. UM: unit of measurement; Fs: sampling frequency.

Dataset	Sensor Orientation			UM	Fs
	x	y	z		
ADL	vertical (downward)	lateral (left)	posterior	$\frac{m}{s^{2}}$	200 Hz
Rempark	anterior	vertical (upward)	lateral (left)	$\frac{m}{s^{2}}$	40 Hz
Daphnet	anterior	vertical (downward)	lateral (right)	mg	64 Hz

**Table 3:** Summary of the features extracted from the inertial signals.

Domain	Feature (# Features per Channel)	Description
Time	Median (1)	Median value
	RMS (1)	Root mean square value
	Range (1)	Range of values
	Min (1)	Minimum value
	Max (1)	Maximum value
	Quantile (2)	25th and 75th quantile values
	Entropy (1)	Shannon entropy
	Increments (1)	Mean value increments
	PCA (3)	PCA coefficients of the first three principal components
	Jerk (1)	Acceleration rate of change
	Sum (1)	Sum of values
Frequency	PosturalBand (1)	Spectral density in the 0–0.7 Hz band
	LocoBand (1)	Spectral density in the 0.7–3 Hz band
	FreezeBand (1)	Spectral density in the 3–8 Hz band
	sEntropy (1)	Shannon spectral entropy
	sPeak (1)	Maximum value of the spectral signal
	Kurtosis (1)	Spectral kurtosis
	Skewness (1)	Spectral skewness
	nHarmonics (1)	Number of harmonics
	pHarmonic (1)	Frequency of the principal harmonic
	wHarmonic (1)	Width of the principal harmonic
	aHarmonic (1)	Area under the principal harmonic

**Table 4:** Summary of the layers and parameters of the CNN model with 1D separable convolution. GAP: global average pooling; f: number of filters; k: filter size; p: pooling size; d: dropout rate; u: number of neurons.

Layer	Layer Parameters	Output Shape	# Parameters
Input	-	(80, 3)	0
1D Separable convolution	f = 100, k = 10	(71, 100)	430
Max pooling	p = 3	(23, 100)	0
1D Separable convolution	f = 40, k = 10	(14, 40)	5040
GAP	-	40	0
Dropout	d = 0.5	40	0
Fully connected	u = 1	1	41
Total trainable parameters			5511

**Table 5:** Performance of the gait recognition algorithms on the ADL dataset. LR: logistic regression; RF: random forest; CNN: convolutional neural network; AUROC: area under the receiver operating characteristic; EER: equal error rate.

Approach	Set	Sensitivity	Specificity	F-Score	AUROC	EER (%)
	Train	0.962	0.909	0.604	0.974	8.7
LR	Validation	0.933	0.933	0.682	0.975	6.7
	Test	0.946	0.896	0.528	0.961	10.1
	Train	1	0.974	0.849	1	2.5
RF	Validation	0.920	0.934	0.676	0.972	6.7
	Test	0.954	0.894	0.526	0.963	10.3
	Train	0.941	0.948	0.704	0.983	5.5
CNN	Validation	0.947	0.956	0.764	0.985	4.6
	Test	0.956	0.929	0.621	0.979	6.1

**Table 6:** Effect of the context algorithms on the Rempark dataset.

Method	Predicted Episodes (Activation Horizon)	Detected Episodes (Activation Latency)	Time Active
Logistic regression	89.0% (7.5 s)	11% (1.4 s)	39.8%
Random forest	92.0% (6.5 s)	8% (0.9 s)	38.4%
1D SepConv CNN	96.0% (8.2 s)	4% (0.7 s)	43.5%
Threshold method	95% (10.1 s)	5% (0.8 s)	39.5%

**Table 7:** Effect of the context algorithms on the Daphnet dataset.

Method	Predicted Episodes (Activation Horizon)	Detected Episodes (Activation Latency)	Time Active
Logistic regression	80.0% (11.0 s)	18% (1.9 s)	42.1%
Random forest	84.0% (11.5 s)	14% (1.1 s)	41.0%
1D SepConv CNN	87.0% (10.5 s)	10% (1.4 s)	45.4%
Threshold method	94% (23.5 s)	2% (1.1 s)	45.2%

**Table 8:** Computational complexity of deep learning-based FoG detection approaches and context algorithms proposed in the present work. CNN: convolutional neural network; LSTM: long short-term memory; FLOPs: floating point operations; K: thousands, M: millions.

Model	# Parameters (K)	# FLOPs (M)	Prediction Time (ms)
CNN-LSTM [23]	288.98	4.76	42
CNN-Transformer [35]	87.82	8.93	45
1D-CNN [32]	43.18	3.14	40
CNN-LSTM [38]	32.96	0.37	38
Multi-head CNN [46]	10.82	0.39	43
1D SepConv CNN	5.51	0.41	39
Threshold method	0.001	0.000772	0.031

## Data Availability

The ADL dataset is available on request from the corresponding author. The Rempark dataset belongs to the Technical Research Centre for Dependency Care and Autonomous Living (CETpD), Universitat Politecnica de Catalunya. The data were collected in the Rempark project and are available under reasonable request from the corresponding owners. The Daphnet freezing-of-gait dataset is available at https://archive.ics.uci.edu/ml/datasets/Daphnet+Freezing+of+Gait (accessed on 24 February 2023).

## Abbreviations

The following abbreviations are used in this manuscript:

ADL	Activities of daily living
AdamW	Adaptive moment estimation with decoupled weight decay
AUROC	Area under the receiver operating characteristics curve
CNN	Convolutional neural network
DL	Deep learning
EER	Equal error rate
FLOPs	Floating point operations per second
FN	False negative
FP	False positive
FoG	Freezing of gait
GAP	Global average pooling
H&Y	Hoehn and Yahr
IMU	Inertial measurement unit
LSTM	Long short-term memory
ML	Machine learning
PD	Parkinson’s disease
PwPD	Patients with Parkinson’s disease
ReLU	Rectified linear unit
RF	Random forest
SMOTE	Synthetic minority oversampling technique
TN	True negative
TP	True positive
UPDRS	Unified Parkinson’s disease rating scale
